# The Effect of Biomaterials Used for Tissue Regeneration Purposes on Polarization of Macrophages

**DOI:** 10.1089/biores.2015.0041

**Published:** 2016-01-01

**Authors:** Geesien S.A. Boersema, Nienke Grotenhuis, Yves Bayon, Johan F. Lange, Yvonne M. Bastiaansen-Jenniskens

**Affiliations:** ^1^ Department of Surgery, Erasmus MC, University Medical Center Rotterdam, Rotterdam, The Netherlands.; ^2^ Department of Orthopedics, Erasmus MC, University Medical Center Rotterdam, Rotterdam, The Netherlands.; ^3^ Department of Otorhinolaryngology, Head and Neck Surgery, Erasmus MC, University Medical Center Rotterdam, Rotterdam, The Netherlands.; ^4^ Metronix-Sofradim Production, Trévoux, France.

**Keywords:** biomaterials, *in vitro* models, macrophage response, review

## Abstract

Activation of macrophages is critical in the acute phase of wound healing after implantation of surgical biomaterials. To understand the response of macrophages, they are often cultured *in vitro* on biomaterials. Since a wide range of biomaterials is currently used in the clinics, we undertook a systematic review of the macrophage polarization in response to these different surgical biomaterials *in vitro*. Beside the chemistry, material characteristics such as dimension, pore size, and surface topography are of great influence on the response of macrophages. The macrophage response also appears to depend on the differences in sterilization techniques that induce lasting biochemical changes or residues of chemicals and their byproducts used for sterilization. Regarding tissue-based biomaterials, macrophages on human or porcine dermis, strongly cross-linked by chemicals elicit in general a proinflammatory response with higher amounts of proinflammatory cytokines. Synthetic biomaterials such as polyethylene, polyethylene terephthalate (PET) + polyacrylamide (PAAm), PET + sodium salt of poly(acrylic acid) (PAANa), perfluoropolyether (PFPE) with large posts, PEG-g-PA, and polydioxanone (PDO) always appear to elicit an anti-inflammatory response in macrophages, irrespective of origin of the macrophages, for example, buffy coats or full blood. In conclusion, in general *in vitro* models contribute to evaluate the foreign body reaction on surgical biomaterials. Although it is difficult to simulate complexity of host response elicited by biomaterials, after their surgical implantation, an *in vitro* model gives indications of the initial foreign body response and allows the comparison of this response between biomaterials.

## Introduction

A wide range of biomaterials are used as implantable medical devices, notably for soft tissue repair. These materials have their own characteristics with regards to composition, mechanical strength, topography, porosity, and chemistry. Implantation of biomaterials is always associated with tissue damage, more or less important, according to the invasiveness of the surgical procedure, that is, surgical treatment of the disease and biomaterial delivery. Initially, the body response most often starts with blood coagulation followed by wound healing. This process is characterized by protein adsorption to the biomaterial, followed by recruitment of cells including macrophages already 60 min after implantation of the material. In response to the cytokines and chemokines produced by the macrophages, cells involved in wound healing are attracted.^1^ The inflammatory response is very important following surgical tissue damage and material implantation, also called foreign body reaction.

Activation of macrophages is critical in the acute phase of wound healing.^2,3^ Macrophages can be roughly divided into proinflammatory macrophages, also called M1 macrophages, and anti-inflammatory macrophages, also called M2 macrophages.^4,5^ The balance between M1 and M2 plays a critical role in the phagocytosis of pathogens, the clearance of apoptotic cells and the healing and remodeling of injured tissues.^6^

Almost immediately after implantation, macrophages are recruited to biomaterials. Depending on the biomaterial specific characteristics, these macrophages will determine the type and intensity of the host response.^6,7^ The eventual success of an implantable medical device strongly depends on this response.

The host response after implantation is inter alia guided by soluble factors such as cytokines and growth factors, as communication agents between cells, active in the wound healing process. Several studies point out the cytokine classification according to their role in the foreign body response.^8–10^ These soluble factors are, among other cell types, produced by macrophages and play pivotal roles in wound healing and serve as useful markers of M1/M2 activation.^7,10–12^

The pivotal role of macrophages in the wound healing process, including tissue repair or regeneration supported by biomaterials, is a strong incentive to interrogate the macrophage response, elicited by biomaterials, in well-defined *in vitro* conditions, with reasonable prediction of the complex foreign body reaction by using simplified single cell approaches. For this purpose, human monocyte-derived macrophages, human monocyte cell lines, mouse bone marrow-derived macrophages, and murine macrophage cell lines are used as culture models. In these models, it is examined whether biomaterials elicit a proinflammatory, anti-inflammatory, prowound healing, or an antiwound healing response by macrophages. These models support the first step to analyze materials before use in the clinic. As nicely reviewed by Sridharan et al.^1^ many different properties of the material influence the polarization of the macrophage, among others the mechanical properties, topography, and surface chemistry. Since many types of biomaterials are used in many different culture models with a large variety of read-out parameters, the purpose of this review was to provide an overview of which biomaterial leads to which response, in particular regarding the differentiation and activation of the macrophages and the associated production of soluble factors.

## Materials and Methods

### Search methods

This systematic review was conducted in accordance to the Preferred Reporting Items for Systematic Reviews and Meta-Analyses (PRISMA) guidelines.

### Search strategy and study selection

On the 29th of June 2015 a systematic literature search was performed using Medline, EMBASE, Cochrane, PUBMED, Google Scholar, and Web-of-Science libraries (Supplementary Appendix S1). There were no restrictions used during the search based on the publication year, publication language, and type of study. Two researchers (G.S.A.B. and N.G.) screened all titles and abstracts of the identified articles independently for their relevance. From all articles that possibly met the inclusion criteria, the full-text version was retrieved and assessed for inclusion. Disagreement was resolved by discussion or requesting advice from a third author (Y.M.B.J.).

An article was eligible for inclusion when it reported on macrophages and their response to biomaterials in an *in vitro* model. Presentations, reviews, and letters to the editor were not included. All references from the selected articles were screened for further possible inclusions.

### Data extraction and analysis

The extracted data are presented in separate tables. The following information was retrieved from each study: first author, year of publication, culture model, biomaterial, and cytokine expression. A meta-analysis could not be performed due to the lack of sufficient comparative studies and the important variability of the *in vitro* macrophage models (e.g., cell origin and isolation procedure, culture conditions, markers).

## Results

### Search

**FIG. 1.**
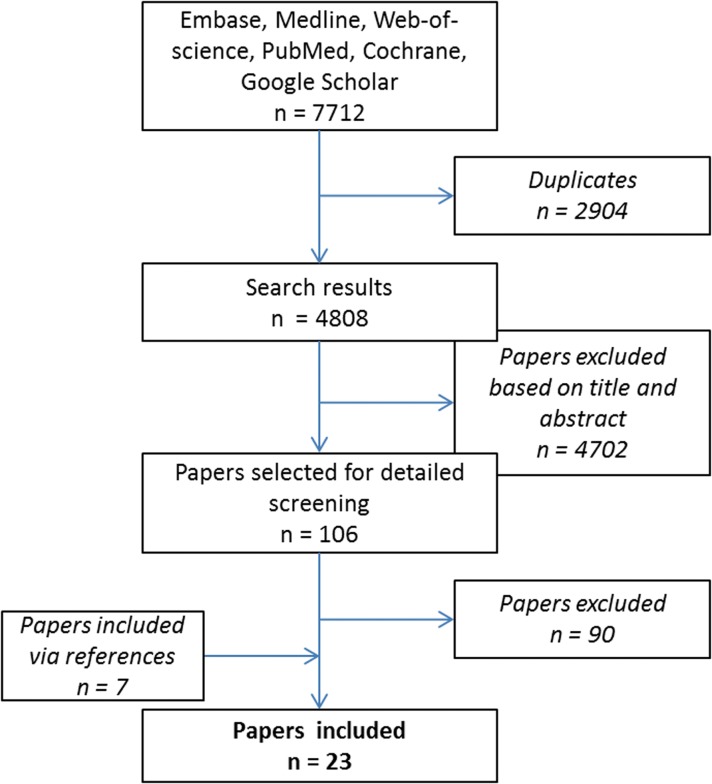
Study selection for relevant articles.

After the exclusion of 2904 duplicates we identified 4275 references. After screening the titles and abstracts, we excluded another 4169 articles. The other 106 articles were regarded relevant and evaluated as full text. After careful reviewing the full text, another 90 were excluded. In addition seven articles were included via references, resulting in 23 included articles (Fig. 1).

### Culture models/experimental conditions

All included studies cultured monocytes or macrophages on biomaterials. However, substantial differences were found in cell culture conditions between the studies. Monocytes isolated from a human buffy coat or human peripheral blood were used in 19/23 of the studies.^7,8,11,13–28^ In the other four studies, one used monocytes derived from mouse bone marrow,^4^ one used the RAW 264.7 cell line (mouse leukaemia monocyte macrophage cell line),^9^ and the other two used the THP-1 human monocyte cell line^10,29^ (Table 1). In most of the studies, no additional factors were added to the culture medium. However, some also added soluble factors to the media. Medium with lipopolysaccharides (LPS) was the most common, but media also contained LPS/interferon gamma (IFN-γ), interleukin (IL)-4, IL-4/IL-13, or monocyte chemotactic protein (MCP)-1/IL-6/IFN-γ. The culture time varied from 2 h to 14 days, but the majority cultured for 1, 3, 7, and/or 10 days.

**Table 1.** **Included Studies Cultured Monocytes or Macrophages on Biomaterials**

Author	Year	Cells
Almeida et al.^13^	2014	Human buffy coat
Ballotta et al.^28^	2014	Human buffy coat
Bartneck et al.^14^	2010	Human peripheral blood
Bartneck et al.^15^	2012	Human peripheral blood
Bhardwaj et al.^16^	2001	Human buffy coat
Bhattacharjee et al.^17^	2013	Human peripheral blood
Bota et al.^18^	2010	Human peripheral blood
Brodbeck et al.^8^	2002	Human peripheral blood
DeFife et al.^19^	1995	Human peripheral blood
Fearing et al.^29^	2014	THP-1 cell line^a^
Garg et al.^4^	2013	Mouse bone marrow-derived Mφ
Gretzer et al.^20^	2003	Human buffy coat
Grotenhuis et al.^7^	2013	Human buffy coat
Jones et al.^21,31^	2007	Human peripheral blood
Oliveira et al.^22^	2012	Human buffy coat
Orenstein et al.^23^	2009	Human peripheral blood
Orenstein et al.^24,25^	2010	Human peripheral blood
Schachtrupp et al.^11^	2003	Human buffy coat
Schutte et al.^10^	2009	THP-1 cell line^a^
Spiller et al.^26^	2014	Human buffy coat
Van den Beucken et al.^9^	2007	RAW 264.7 & J744A.1^b^
Wagner et al.^27^	2003	Human peripheral blood

^a^ THP human leukemic monocyte.

^b^ RAW/J744 murine macrophage cell line.

### Biomaterials

Biomaterials can be divided into three groups namely the nonbiodegradable polymers (synthetic), biodegradable polymers (synthetic), and biologic materials.^30^ In total 35 different materials were used in the included articles (Table 2).

**Table 2.** **Reviewed Biomaterials and Their Predominant Reaction**

Biomaterial	Predominant reaction of macrophages in contact with biomaterial	Low/high cytokine production	Refs.
PTFE	Mainly proinflammatory	High	^10,15^
ePTFE	Proinflammatory and anti-inflammatory	High/high	^9,17,25^
PET	Mainly proinflammatory	High	^6,7,20^
PET + BDEDTC	Mainly proinflammatory	High	^7,20^
PET + BDEDTC + PAAm	Mainly anti-inflammatory	High	^7,20^
PET + BDEDTC + PAANa	Mainly anti-inflammatory	High	^7,20^
PET + BDEDTC + DMAPAAmMeI	Mainly proinflammatory	High	^7,20^
Parietex™ Composite	Proinflammatory and anti-inflammatory	High/high	^6^
Polyethylene	Mainly anti-inflammatory	Low	^9,18^
Polyurethane	Proinflammatory and anti-inflammatory	High/high	^9,15,18^
PFPE (small posts)	Mainly proinflammatory	High	^13^
PFPE (large posts)	Mainly anti-inflammatory	High	^13^
PP	Proinflammatory and anti-inflammatory	Low/low	^6,26^
PP + polyglactin	Mainly proinflammatory	High	^10^
Poly(ethylene glycol):poly(acrylate)	Mainly anti-inflammatory	Low	^27^
Poly-d-lysine-PAH	Mainly proinflammatory	Low	^8^
Silicone	Proinflammatory and anti-inflammatory	High/high	^15^
Polylactic acid	Proinflammatory and anti-inflammatory	High/high	^12^
Poly(ethylene oxide)	Mainly proinflammatory	High	^14^
Bio-A	Mainly proinflammatory	Low	^23^
Polydioxanone	Mainly anti-inflammatory	High	^3^
Poly-e-caprolactone bisurea	Mainly anti-inflammatory	High	^27^
Poly(urethane urea)	Proinflammatory and anti-inflammatory	Low/low	^19^
Collamend™	Mainly proinflammatory	High	^24^
Permacol™	Mainly proinflammatory/proinflammatory and anti-inflammatory	High/low	^6,24^
Allomax	Mainly proinflammatory	High	^22,23^
FlexHD	Mainly proinflammatory	High	^22,23^
Alloderm	Mainly proinflammatory	Low	^22,23^
Strattice™	Mainly proinflammatory	Low	^24^
Surgisis^®^	Mainly proinflammatory	Low	^24^
Collagen coating	Mainly proinflammatory	High	^28^
Ultrafoam	Mainly proinflammatory	Low	^16,25^
Silk	Mainly proinflammatory	High	^16^
Keratin	Proinflammatory and anti-inflammatory	Low/high	^28^
Chitosan	Proinflammatory and anti-inflammatory	Low/high	^12,21^

This table shows results coming from different macrophage models, not necessarily equivalents. The results are adapted generally from one study.

BDEDTC, poly(styrene-co-benzyl N,N-diethyldithiocarbamate); DMAPAAmMeI, methyl iodide of poly[3-(dimethylamino)propyl]acrylamide; ePTFE, expanded polytetrafluoroethylene; PAAm, polyacrylamide; PAANa, sodium salt of poly(acrylic acid); PFPE, perfluoropolyether; PET, polyethylene terephthalate; PP, polypropylene; PTFE, polytetrafluoroethylene.

### Nonbiodegradable synthetic polymers

Expanded polytetrafluoroethylene (ePTFE) and polytetrafluoroethylene (PTFE) are commonly applied hernia mesh and vascular grafts materials. PTFE, also known as Teflon^®^, is naturally hydrophobic and nonporous. ePTFE is stretched and nano-porous and was introduced under the trademark GORE-TEX^®^, in 1969. Monocytes (precursors of macrophages) on PTFE produced low amounts of IL-1β and high amounts of tumor necrosis factor (TNF)-α and IL-6 in the first days of culture. IL-10 levels increased during culture time, it was mainly produced between culture day 2 and 6.^11,16^ After a culture time for 8–10 days the production of TNF-α and IL-10 decreased, while IL-8 increased after 8 days of culture.^16^ Granulocyte-macrophage colony-stimulating factor (GM-CSF) was secreted during the whole culture time (1–10 days).^11,16,18^ Macrophages on PTFE also produced platelet-derived growth factor-BB, and matrix metalloproteinase 9 but vascular endothelial growth factor (VEGF) was undetectable.^26^ Macrophages on ePTFE produced more proinflammatory cytokines (IL-1α, IL-1β, IL-6, and TNF-α) and chemokines (MCP-1, MIP1-β, and MCP-3) in association with an increase of the pore size of the material.^18^ In contrast, immortalized human monocyte cell line (THP-1) cultured on ePTFE induced an anti-inflammatory and prowound healing profile characterized by a high IL-10 production in another study.^10^

Current surgical applications of polyethylene terephthalate (PET) are that is, surgical meshes, vascular grafts, heart valves, and sutures. Macrophages on PET produce predominantly proinflammatory cytokines, MCP-3, TNF-α, IL-6, IL-1β, MIP-1α,^7,8,31^ and proinflammatory chemokine IL-8.^31^

PET is also used in combination with different “coatings.” These coatings affect biomaterial adherent monocyte/macrophage cytokine expression through modification of surface chemistry. Different coatings are used: PET + poly(styrene-co-benzyl N,N-diethyldithiocarbamate) (BDEDTC; hydrophobic), PET + BDEDTC + polyacrylamide (PAAm; hydrophilic and neutral), PET + BDEDTC + sodium salt of poly(acrylic acid) (PAANa; hydrophilic and anionic), PET + BDEDTC + methyl iodide of poly[3-(dimethylamino)propyl]acrylamide (DMAPAAmMeI; hydrophilic and cationic), and PET + absorbable, continuous and hydrophilic collagen film (Parietex™ Composite). Macrophages on PAAm and PAANa surfaces reacted anti-inflammatory with a higher IL-10 production and lower IL-8 production than when cultured in PET without coating during the culture time from day 3 till day 10.^8,31^ Monocytes adherent to PAAm produced the most IL-6, IL-1β, IL-10, IL-8, and MIP-1β at all time points, compared to the other coatings in combination with PET.^31^ Macrophages cultured for 3 to 7 days, produced the highest concentrations of IL-1β on PAAm and least on BDEDTC. MIP-1β concentrations were greatest with PAANa at day 3. DMAPAAmMeI promoted a decrease of IL-10 and IL-1RA in macrophages, but it did not influence the expression levels of IL-1β, TNF-α, and IL-6.^7,29^ BDEDTC, PAAm, PAANa, and DMAPAAmMeI let the IL-1β, TNF-α, and IL-6 expression levels relatively unchanged at the end of culture time.^8,31^ Parietex Composite (Covidien) induced high levels of proinflammatory and anti-inflammatory proteins.^7^

Macrophages cultured on polyethylene (PE), with versatile use such as catheters and joint prosthesis, produced low amounts of cytokines in general but the balance was more toward anti-inflammatory and prowound healing cytokines.^10,19^ Both THP-1 cell line monocytes/macrophages and macrophages isolated from human buffy coats cultured on polyurethane (PU), often used in blood contact applications, produced high levels of anti-inflammatory and prowound healing cytokines.^10,16^ Perfluoropolyether (PFPE) is a nondegradable homopolymer that shows chemical inertness, lipophobicity, and has very low surface energy.^14^ This material was tested with different micro topographies and the effect on the response of macrophages. Different surface topographies resulted in different cytokine production by macrophages. An M1 surface marker, 27E10, had an enhanced expression in response to closely packed small posts, comparable to when macrophages were stimulated with LPS. In contrast, macrophages cultured on PFPE with large posts expressed the M2 surface marker CD163 the most. Large posts also resulted in significantly the highest M2-M1 index based on macrophages surface markers.^14^

Poly(propylene) (PP) is also commonly used mesh and suture materials in surgery. Both an anti-inflammatory reaction characterized by high levels of CCL-18 and IL1-RA among others and a proinflammatory reaction characterized by production of IL-8, IL-6, and IL-1β by macrophages seeded both for 24 h or 3 days on PP were observed.^7,27^ When combined with polyglactin 910 materials (Vypro II^®^; Ethicon), monocyte/macrophages also released high amounts of TNF-α, IL-6, and low amounts of IL-10 after 5 days of culture, which indicates a proinflammatory response.^11^

Poly(ethylene glycol):poly(acrylate) PEG-g-PA is also modified with cell adhesion promoting peptides (YRGDS and YEILDV, peptides recognized by integrins) to modulate the host cell response.^27^ Culturing macrophages on PEG alone resulted in low production of TNF-α, IL-1β, IL-6, and IL-8. Macrophages on peptide modified PEG-*g*-PA produced even lower levels of TNF-α and IL-6.^27^

Poly-d-lysine (PDL) and poly(allylamine hydrochloride) (PAH), both synthetic polymers, were coated with DNA and seeded with two different cell line macrophages. All experiments showed decreased levels of TNF-α compared with the cultured polymers with LPS-stimulated murine macrophages (density of 1 × 10^5^ cells/cm^2^).^9^ The cytokine secretion of IL-1β, IL-10, and TGF-β1 was not different between macrophages cultured on PDL and PAH with or without LPS stimulation.^9^ Monocytes on silicone cultured for 10 days produced high GM-CSF and IL-8.^16^ TNF-α and IL-10 were produced at high levels the first 2–6 days, where after the production decreased.^16^

### Biodegradable synthetic polymers

Synthetic biodegradable polymers were first used as biodegradable sutures in the 1960s. Synthetic biodegradable implants are mostly used in the clinic as soft/hard tissue reinforcement materials or temporary barriers/wound supports. Their purpose is to avoid a chronic foreign body reaction.^32^ These polymeric biomaterials are based on lactic acid and glycolic acid, and other monomers, including dioxanone and trimethylene carbonate e-caprolactone as homopolymers and copolymers.

Polylactic acid (PLA) induces production of IL-6, IL-12/23, and IL-10, these cytokines are both proinflammatory and anti-inflammatory, it appeared like human monocytes cultured on PLA exhibited a heterogeneous profile.^13^

Poly(D,L-lactide-co-glycolide) (PLGA) represents a major class of materials widely used in surgical applications and tissue engineering.^15^ Bartneck et al. generated 3D nano-fibrous meshes in different porosities PLGA/sP(EO-stat-PO) and a 2D NCO-sP(EO-stat-PO) hydrogel. NCO-sP(EO-stat-PO) and sP(EO-stat-PO) are ethylene oxide-derived polymers, used for preventing unspecific protein adsorption and cell adhesion.

Macrophages on the 2D materials formed clusters with an elevated release of IL-1β and TNF-α. Macrophages produced more IL-8 and CCL-4 (proangiogenic chemokines) on the more covered 3D nanofibers PLGA/sP(EO-stat-PO).^15^

Macrophages seeded on a copolymer of glycolic acid and trimethylene carbonate, also known as GORE^®^ BIO-A^®^ Tissue Reinforcement (WL Gore Assoc), produced very low proinflammatory cytokine levels.^24^ Polydioxanone (PDO) polymer is developed for biodegradable wound closure sutures. Bone marrow-derived macrophages were cultured on different PDO diameter fibers and pore sizes. An increase of the fiber/pore size resulted in an increased expression of anti-inflammatory and angiogenic markers as VEGF, TGF-β, and FGF2.^4^

The impact of mechanical cues on adherent monocytes on poly-e-caprolactone bisurea (PCL-U4U) was investigated. It has been demonstrated that strain affects macrophage response in terms of signaling and differentiation. Moderate strain (7%) elicits polarization toward a reparative M2 profile and enhance the expression of genes participating in the immune response.^28^

Poly(urethane urea) elicited very small amounts of TNF-α and IL-10.^20^

### Biologic materials

Biologic materials are either decellularized tissues such as human or porcine skin or porcine small intestine submucosa (SIS), or fabricated scaffolds or meshes made of natural molecules such as collagen, chitosan, silk, or keratin. The decellularized tissues can have additional chemical cross-links to alter the degradation speed.^33^

After 7 days of culture CollaMend™ FM Implant (Bard/Davol), a moderately chemically cross-linked porcine dermis, mostly elicited a proinflammatory response in macrophages with high IL-1β, IL-6, IL-8, and VEGF production.^25^ Macrophages on Permacol™ (Covidien), a slightly chemically cross-linked porcine dermal matrix, produced high IL-1β, IL-6, IL-8, and VEGF levels after 7 days of culture.^25^ But in other settings, low levels of both proinflammatory and anti-inflammatory proteins after 3 days of culture, were released by macrophages, in the presence of Permacol.^7^ There were no differences in culture method between the two studies. AlloMax™ Surgical Graft (Bard/Davol) and FlexHD^®^ (Ethicon), nonchemically cross-linked decellularized dermis but of human instead of porcine origin, also induced mainly proinflammatory reactions with high IL-1β, IL-6, IL-8, and VEGF cytokine production.^23,24^ AlloDerm^®^ Regenerative Tissue Matrix (LifeCell) (nonchemically cross-linked decellularized human dermis) induced a lower proinflammatory response than the other decellularized human dermis, characterized by lower expression of IL-1β, IL-6, IL-8, and VEGF.^23,24^ Macrophages seeded on the noncross-linked porcine dermis, Strattice™ (LifeCell), or on the noncross-linked porcine SIS, Cook^®^ Biodesign^®^ Surgisis^®^ (Cook), produced low levels of IL-1β, IL-6, IL-8, and VEGF.^25^

Macrophages cultured on collagen coatings expressed mostly M1 surface markers (CD86^+^) and express both M1 and M2 markers.^29,34^ These macrophages produced also high levels of proinflammatory cytokines. Another collagen-based biomaterial is Avitene™ UltraFoam™ Collagen Sponge (Bard/Davol; bovine source collagen sponge). Macrophages cultured on this gel did not produce IL-1β, and IL-6 production was only seen at day 1 and was lower produced at day 3, indicating that the response of the macrophages was not proinflammatory.^17^

Other noncommercial biopolymers have been investigated. Bhattacharjee et al. studied the macrophage responses against silk-fibroin and silk-sericin-based 2D films, and 3D silk-fibroin scaffolds.^17^ These scaffolds are used for tissue engineering and drug delivery. The 3D fibroin scaffold induced gene expression of proinflammatory genes and accordingly the production of IL-1β and IL-6. Silk-sericin films also induced IL1-β gene expression.^17^

Two other biologic biomaterials are keratin and chitosan. Keratin has been described for applications such as tissue regeneration, hemostasis, and wound healing. A low foreign body reaction against keratin was described characterized with predominantly M2 (CD206^+^) macrophages, high levels of IL-10, and low levels of IL-1β and IL-6.^29^ Chitosan (a natural polysaccharide composed of randomly distributed β-(1–4)-linked d-glucosamine and N-acetyl-d-glucosamine) induced an M2 phenotype in one study based on low TNF-α that decreased with time and high IL-10 and TGF-β1 levels cytokines.^22^ In another study chitosan induces a predominant M1 response based on high production of TNF-α and IL-12/IL-23 and low expression of IL-6, especially in the 3D geometry.^13^ Oliveira et al. cultured on chitosan films instead of 3D geometry.^13^

## Discussion

Macrophages are key components of tissue repair and remodeling in wound healing. Their polarization appears to depend on the type of biomaterial and their characteristics. The release of a variety of cytokines and chemokines is decisive for the differentiation and activity of monocytes.^35^ Here, we reviewed the macrophage response on different materials *in vitro* used in tissue repair and regeneration and provided an overview of commonly seen macrophage responses to these biomaterials.

Based on the literature review, we have shown that the dimensions of the cultured material is of great influence on the response of macrophages. This was (mostly) investigated in PFPE, ePTFE, chitosan, and PDO. The association was, however, different between increasing fiber/pore size and the polarization or release profile of macrophages. Two synthetic biomaterials showed the opposite effect of pore size. Bartneck et al. showed a higher proinflammatory effect when the pore size was smaller in PFPE.^14^ Bota et al. saw a higher proinflammatory effect of macrophages cultured on ePTFE when the pores are larger.^18^ Almeida et al. saw the same effect, on scaffolds based on chitosan, a biologic material.^13^ In contrast, Garg et al. cultured macrophages on PDO, a synthetic biodegradable material, and they showed that large pores induced M2 phenotype and a decreased M1-marker expression. However, in this study, mouse bone marrow-derived macrophages were used instead of human macrophages. In an *in vivo* study with biodegradable pHEMA (2-hydroxyethyl methacrylate) hydrogel scaffolds it was also shown that pore size affect macrophage response. Pore size of 34 μm was shown to reduce fibrous encapsulation, however, more M1 cells were found than at those scaffolds with a larger pore size of 160 μm, this indicate that the initial M1 response is necessary.^36^

As expected, macrophages on moderately chemically cross-linked human or porcine dermis responded in general proinflammatory with higher amounts of proinflammatory cytokines than the macrophages cultured on nonchemically cross-linked or slightly chemically cross-linked materials. This was also seen in *in vivo* studies were Collamend™ FM Implant (Bard/Davol) induced a chronic foreign body response and downstream encapsulation.^37,38^ This mainly proinflammatory response lead to chronic fibrosis.^39^ Unfortunately, in all *in vitro* studies on these biologic materials, only investigated IL-1β, IL-6, IL-8, and VEGF, known for their mainly proinflammatory response, no anti-inflammatory cytokines were measured. A recent review presented that moderately to strongly cross-linked collagen materials can alter normal wound healing. In particular, residues of chemical cross-links in the material were associated with a M1 macrophage response, and inhibition of M2 macrophage polarization.^33^

Chitosan, another biopolymer, showed a predominant M1 response with a very low IL-6 production.^13^ The same effect was seen on the collagen gel; mainly proinflammatory cytokines were produced, but no production of IL-1β.^17^ This can be considered a pleiotropic function of IL-6 and IL-1β. It is known that IL-6 can act either proinflammatory or anti-inflammatory, depending on the environment.^40^ IL1-β is a key cytokine that is important for wound healing, activating and recruiting fibroblasts, resulting in expression of extracellular matrix components like collagen, elastin, and glycosaminoglycans.^41–43^

Some materials induced different responses in different experiments such as acellular human dermis from different companies. This could be due to the differences in sterilization techniques that induce lasting biochemical changes or residues of the chemical used for sterilization; gamma radiation is used for AlloMax Surgical Graft (Bard Davol); FlexHD (Ethicon) is sterilized by detergents, disinfectants, and ethanol; and the sterilization process of AlloDerm Regenerative Tissue Matrix is proprietary. AlloDerm induced the least of the proinflammatory cytokines. Also, the methods of decellularization and processing of the materials were different, which can be an additional explanation for the different foreign body responses, notably explained by chemical residues, used for decellularization and fat removal.

Comparing all the responses of the different materials, it appears that polyethylene, PET + PAAm, PET + PAANa, PFPE (large posts), PEG-g-PA, and PDO always elicited an anti-inflammatory response in macrophages, irrespective of origin of the macrophages.

*In vitro* testing of macrophage response to biomaterial can be an initial means of assaying biocompatibility. Macrophages are certainly great drivers of the acute inflammation reaction. Neutrophils (polymorphonuclear leukocytes [PMNs]) also characterize acute inflammatory response. Mast cell degranulation with histamine release and fibrinogen adsorption is also known to mediate acute inflammatory responses to implanted biomaterials.^44,45^ For a complete *in vitro* model, these factors should also be taken into account. For example, Surgisis is known to strongly activate PMNs, particularly neutrophils.^46^ Bryan et al. show a strong release of Reactive Oxygen Species by Surgisis versus Alloderm and Permacol, in animal models.^47^

In general, *in vitro* models are useful in the first step to evaluate the foreign body reaction on surgical biomaterials. Although it is difficult to simulate the environment during a surgical procedure, an *in vitro* model gives an indication of the initial foreign body response even in an environment that simulates an infection by, for instance, addition of LPS. Grotenhuis et al. proved this by simulating a bacterial infection in their *in vitro* model, but the macrophage response remained biomaterial dependent.^48^ In this perspective it will be useful to test, for example, other surgical biomaterials like tissue adhesives that are used in the clinic.

Because of the complexity of host response to foreign body material it is difficult to predict the *in vivo* outcome from *in vitro* assays. Wolf et al. developed an *in silico* analysis by using an *in vitro* assay that characterized the dynamic inflammatory response of human monocyte-derived macrophages to biomaterials in combination with quasi-mechanistic analysis.^49^ This approach can be used to better predict the *in vivo* response. More sophisticated systems, like multicellular approaches combining macrophages with fibroblasts, endothelial cells, and stem cells, aiming at recreating a better mimicking system, should certainly be useful for the in-depth investigation of the behavior of materials *in vivo.*^50^

Simple models as single cell approaches should be used for screening approaches, enabling the direct comparison of materials. Macrophage models can gain even higher interest by including monocytes from specific patient groups, like obesity, which may react differently to materials.

## Conclusion

With this review, we provided an overview of *in vitro* responses of macrophages to many different biomaterials. Some materials performed comparable in different studies and it appears clear which response these biomaterials elicit in macrophages. Other materials behaved differently in different culture setups. Therefore, all physical properties (e.g., stiffness, pore size, strain, topography, and surface chemistry) of the biomaterial must be announced, because these features can induce different macrophage behavior.^1,39^ Each step in cell culture is critical, the macrophage isolation, activation of the macrophage before culture or not, time duration of cell culture since it conditions the phenotype/differentiation of cells, and the type of culture medium, minimal changes in culture methods can cause the different outcome.^2,35,51,52^ *In vitro* culture models using macrophages on biomaterials are a valuable addition to the development of new biomaterials. Based on this review there is, however, a need for standardized culture models and a systematic comparison to the *in vivo* response.

## Supplementary Material

Supplemental data

## Abbreviations Used

BDEDTC: poly(styrene-co-benzyl N,N-diethyldithiocarbamate)
DMAPAAmMeI: methyl iodide of poly[3-(dimethylamino)propyl]acrylamide
ePTFE: expanded polytetrafluoroethylene
GM-CSF: granulocyte-macrophage colony-stimulating factor
IFN-γ: interferon gamma
IL: interleukin
LPS: lipopolysaccharides
MCP: monocyte chemotactic protein
PAAm: polyacrylamide
PAANa: sodium salt of poly(acrylic acid)
PAH: poly(allylamine hydrochloride)
PDL: poly-d-lysine
PDO: polydioxanone
PFPE: perfluoropolyether
PLA: polylactic acid
PLGA: poly(D,L-lactide-co-glycolide)
PMNs: polymorphonuclear leukocytes
PP: polypropylene
PTFE: polytetrafluoroethylene
SIS: small intestine submucosa
TNF: tumor necrosis factor
VEGF: vascular endothelial growth factor
