# Supplementary material for: The Effect of Biomaterials Used for Tissue Regeneration Purposes on Polarization of Macrophages
Source: Biores Open Access. 2016 Jan 1;5(1):6–14. doi: 10.1089/biores.2015.0041 (PMC4744891; doi:10.1089/biores.2015.0041)
Supplement: Supplemental data [file Supp_Appendix1.pdf]

## Supplementary Appendix S1

We used the following search strategy in the Embase, and the searching strategy was modified in other databases accordingly.

('tissue adhesive'/exp OR 'adhesive agent'/de OR 'surgical mesh'/de OR 'surgical equipment'/de OR 'tissue scaffold'/de OR biomaterial/de OR (adhesive* OR glue* OR bioglue* OR tachocomb* OR bucrilate* OR enbucrilate* OR cyanoacryl* OR mesh* OR 4DDOME OR AIGISRx OR AlloDerm OR AlloMax OR 'Bard Composix EX patch' OR 'BIO-A Tissue Reinforcement prosthesis' OR CollaMend OR DermaMatrix OR DualMesh OR 'Evolution P3EM' OR FasLata OR FlexHD OR FortaGen OR 'IntePro Lite' OR InteXen OR NEOVEIL OR 'Optilene Mesh LP' OR 'Parietex composite' OR Pelvicol OR Pelvisoft OR Pelvitex OR PerFix OR 'Peri-Strips Dry' OR PeriGuard OR Permacol OR Physiomesh OR Strattice OR Surgisis OR TIGR OR Timesh OR 'TiMESH light' OR Tutomesh OR Tutopatch OR Ultrapro OR Ventralex OR Veritas OR Vivosorb OR Vypro OR X-Repair OR XenMatrix OR scaffold* OR biomaterial* OR biocompatib* OR Hemocompatib* OR Haemocompatib* OR resorbable OR (implant* NEAR/3 integrat*)):ab,ti) AND ('macrophage culture'/de OR 'monocyte culture'/de OR ((macrophage/exp OR 'macrophage activation'/de OR monocyte/de OR (macrophag* OR monocyte*):ab,ti) AND ('in vitro study'/exp OR monoculture/de OR ('in vitro' OR culture* OR monocultur*):ab,ti))) NOT ([Conference Abstract]/lim OR [Conference Paper]/lim OR [Review]/lim OR [Conference Review]/lim OR [Letter]/lim OR [Note]/lim OR [Editorial]/lim) AND [english]/lim
